# Characterizing treatment interruptions in the OPERA cohort and virologic outcomes after resumption with bictegravir/emtricitabine/tenofovir alafenamide

**DOI:** 10.1186/s12981-025-00769-x

**Published:** 2025-07-21

**Authors:** Karam Mounzer, Michael D. Osterman, Laurence Brunet, Ricky K. Hsu, Gerald Pierone Jr., Jennifer S. Fusco, Neia Prata Menezes, Joshua Gruber, Travis Lim, Megan Dunbar, Gregory P. Fusco

**Affiliations:** 1 Philadelphia FIGHT Community Health Centers, Philadelphia, PA USA; 2 Epividian, Inc., Raleigh, NC 27601 USA; 3 AIDS Healthcare Foundation and NYU Langone Medical Center, New York, NY USA; 4 Whole Family Health Center, New York, NY USA; 5 Gilead Sciences, Inc., Foster City, CA USA

**Keywords:** Treatment interruption, Unplanned interruption, Resumption, Treatment gap, Antiretroviral therapy, Cohort, HIV, Bictegravir/emtricitabine/tenofovir alafenamide, B/F/TAF, Virologic failure

## Abstract

**Background:**

Despite advancements in antiretroviral therapy (ART) for people with HIV, barriers to adherence remain, potentially leading to long-term gaps in adherence known as treatment interruptions. These treatment interruptions are associated with viral rebound and can impact the effectiveness of the subsequent regimen and the long-term health of the individual. We aimed to characterize unplanned ART treatment interruptions in the OPERA^®^ cohort and investigate virologic outcomes among individuals who resumed treatment with bictegravir/emtricitabine/tenofovir alafenamide (B/F/TAF).

**Methods:**

We identified adults with HIV-1 who were active in care and on an oral ART regimen with ≥ 2 antiretrovirals, including ≥ 1 anchor agent, between 30JUN2021 and 31AUG2023. Individuals with ≥ 1 period of ≥ 45 days without any ART, based on supply from last prescription, were considered to have experienced a treatment interruption. Individuals who resumed treatment by 31AUG2023 were defined as having experienced a treatment interruption with resumption. Each interruption observed during the study period was described, allowing for multiple interruptions per person. Treatment interruptions, pre-interruption regimens, and post-interruption regimens were described. Among individuals who resumed treatment with B/F/TAF, virologic outcomes were investigated through 29FEB2024 using Kaplan-Meier methods. All analyses were repeated with treatment interruption definitions of ≥ 60 and ≥ 90 days.

**Results:**

Of 76,883 people for whom a treatment interruption could be observed, 8,550 (11%) experienced ≥ 1 period of ≥ 45 days without any ART. By 31AUG2023, 4,163 (49%) individuals resumed treatment (mean: 1.25 per person) and were included in the study population. The median age was 44 years, 81% were male, 52% Black, 41% White, and 18% Hispanic. Median time since HIV diagnosis was 118 months. B/F/TAF was the most common pre- and post-interruption regimen (49% and 51%, respectively). The cumulative probability of achieving virologic suppression on B/F/TAF was 68% (95% CI: 57, 78) when the viral load measurement was ≥ 200 copies/mL at resumption.

**Conclusions:**

Treatment interruptions occurred in 11% of ART users in routine clinical care during the 26-month study period. Despite treatment interruption increasing the risk for viral rebound, most individuals who resumed treatment with B/F/TAF were able to achieve virologic suppression or avoid virologic failure.

**Supplementary Information:**

The online version contains supplementary material available at 10.1186/s12981-025-00769-x.

## Background

Advancements in antiretroviral therapy (ART) have extended the lifespan of people with HIV and improved their quality of life [1, 2]. However, there are still many barriers to adherence and engagement in care, including behavioral and psychosocial challenges, language and literacy barriers, individual perceptions, medication-taking skills, structural issues, and polypharmacy [3–7]. These challenges are further exacerbated by the necessity of consistent treatment over the course of a lifetime. Single-tablet regimens have emerged as one option to improve adherence due to their ease of use [8]. A recent review [3] of 31 studies, including predominantly clinical trials, demonstrated significantly higher ART adherence for people with HIV using single-tablet regimens compared to those using multi-drug regimens.

Increased engagement in HIV care has been associated with improved health outcomes and decreased mortality [9–13]. Long-term gaps in adherence, known as unplanned treatment interruptions, can lead to viral rebound and treatment failure which are associated with an increased risk of worsening immune function, poor clinical outcomes, HIV transmission, and the potential for the development of drug resistance [14–17]. This can impact the effectiveness of the next regimen and the long-term health of the individual. Although individuals are at an increased risk of viral rebound during a treatment interruption, they have generally been successful at maintaining virologic control after return to treatment with proper monitoring following treatment interruptions ranging from a couple days to a year [4, 18]. However, there is increased concern about developing resistance among individuals with known adherence issues, leading to a preference for use of antiretrovirals (ARVs) with a high barrier of resistance [7, 19, 20].

In real-world settings, recent studies have found that the latest generation of ART has succeeded in being both durable and forgiving for short-term treatment interruptions while also being generally tolerable for many people with HIV [19, 21–24]. Bictegravir/emtricitabine/tenofovir alafenamide (B/F/TAF), a second-generation integrase strand transfer inhibitor (INSTI), is among the most common ART regimens in the US and is a recommended initial regimen for most people with HIV [7]. However, evidence of the impact of resuming treatment with B/F/TAF after a longer treatment interruption is still limited and is particularly deficient in the US. Given the limited understanding of the frequency and effects of treatment interruptions in real-world settings, this study aimed to characterize oral ART treatment interruptions of ≥ 45 days. Further, this study described the virologic outcomes of individuals who experienced a treatment interruption and resumed treatment with B/F/TAF.

## Methods

### Study population

This study used prospectively collected electronic health records (EHR) data from the Observational Pharmaco-Epidemiology Research and Analysis (OPERA^®^) cohort. In OPERA, the EHR data are the source of all laboratory tests, diagnoses, comorbidities, demographic data, and clinical observations. At the time of the study, OPERA included data from 97 clinics, including large metropolitan multidisciplinary health centers, rural clinics, wellness centers, sexual health clinics, health departments, and federally qualified health centers, located across 21 US states and Puerto Rico. Approximately 14% of all people with HIV in the US were linked to care in OPERA [25]. OPERA complies with all HIPAA and HITECH requirements and has received annual institutional review board (IRB) approval by Advarra IRB, including a waiver of informed consent and authorization for use of protected health information.

**Fig. 1:**
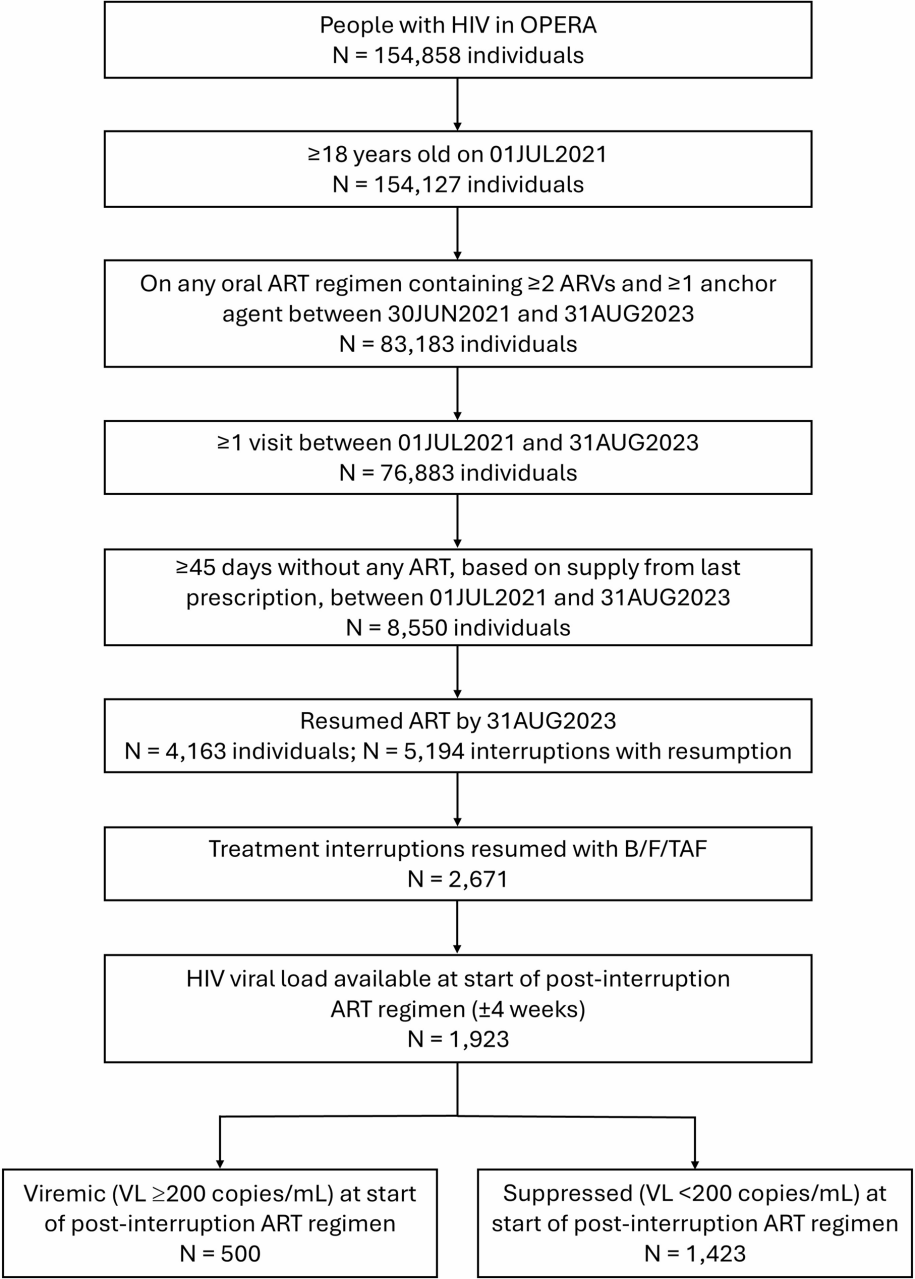
Inclusion in the study population and number of treatment interruptions. ART, antiretroviral therapy; ARV, antiretroviral; N, number

All adults with HIV-1 in OPERA who were ≥ 18 years old on 01JUL2021 were eligible for inclusion if they had ≥ 1 active healthcare visit and were on any oral ART regimen containing ≥ 2 ARVs (excluding boosting agents), including ≥ 1 anchor agent, between 30JUN2021 and 31AUG2023. These individuals represent the group for whom a treatment interruption of ≥ 45 days could be observed. Individuals who experienced both ≥ 45 days without any ART medication, based on supply from last prescription, and resumption of ART by 31AUG2023 were considered to have experienced a treatment interruption with resumption and were included in the study population (Fig. 1).

After experiencing a treatment interruption with resumption, the individual was followed until the first of the following events for each treatment interruption: (1) virologic outcome of interest, (2) change in baseline ART regimen anchor agent or additional treatment interruption, (3) death, (4) loss to follow-up defined as 12 months after last clinical contact, or (5) study end (29FEB2024). If follow-up for a specific treatment interruption with resumption was stopped due to virologic outcome of interest, change in baseline ART regimen anchor agent, or additional treatment interruption, the individual was eligible for additional treatment interruptions during the study period with a separate follow-up period.

### Measurements

A treatment interruption was defined as ≥ 45 days without any ART medication, based on supply from last prescription. This duration was chosen based on previously established algorithms that account for a level of uncertainty around regimen stop dates, reasons for regimen change, date of refills, use of medication samples, and potential stockpiling of excess pills over time [26–28]. Treatment was resumed during the study period with prescription of any ART regimen following the interruption. The start date of a treatment interruption was the earliest between the end of the theoretical supply of an ART prescription or the regimen end date updated by the provider in the EHR. The end date of a treatment interruption was the day before the start of the next ART prescription. Interruptions of long-acting injectable ART regimens were not considered. The pre-interruption regimen and post-interruption regimen corresponded to the ART regimens directly preceding and following the treatment interruption, respectively.

Viral load measurements at interruption were measured within 6 months before the start of the treatment interruption. Viral loads at resumption were measured within 4 weeks before or after the date of resumption. At resumption, viral load measurements were considered to be either virologically suppressed (viral load < 200 copies/mL) or viremic (viral load ≥ 200 copies/mL). The virologic outcomes of interest included virologic failure and virologic suppression. Virologic failure was defined as either (a) two consecutive viral loads ≥ 200 copies/mL or (b) change in anchor agent following a viral load ≥ 200 copies/mL. Virologic suppression was achieved at the first viral load < 200 copies/mL after resumption of ART.

### Statistical analyses

The frequency and duration of treatment interruptions with resumption were described. Additionally, the frequency of treatment interruptions with resumption during each of the first and second year of the study period were described to investigate the potential for lingering effects of the COVID-19 pandemic on healthcare utilization. Descriptive analyses were conducted for all baseline characteristics, pre-interruption regimens, and post-interruption regimens. If an individual experienced multiple treatment interruptions during the eligibility period, each of their pre- and post-interruption ART regimens were counted separately. Absolute numbers and proportions were provided for categorical variables; medians and interquartile ranges (IQRs) were provided for continuous variables.

Virologic outcomes for treatment interruptions resumed with B/F/TAF were investigated according to the viral load at time of resumption of treatment. When a viral load at resumption was unavailable, the treatment interruption was excluded for these analyses. Among resumptions for which the viral load was virologically suppressed at resumption, the virologic outcome of interest was virologic failure. Among resumptions for which the viral load at resumption was viremic, the virologic outcome of interest was achievement of virologic suppression. Time to reach the outcome was assessed using Kaplan-Meier methods with the number at risk considered as the number of treatment interruptions. Cumulative probabilities with corresponding 95% confidence intervals were estimated at 6, 12, and 18 months after resumption of treatment in addition to over the entire follow-up available. An individual was eligible to experience additional treatment interruptions if follow-up for a treatment interruption with resumption was stopped due to virologic outcome of interest, change in baseline ART regimen anchor agent, or additional treatment interruption. Each additional treatment interruption was considered to have a separate follow-up period.

### Sensitivity analyses

Two additional definitions of treatment interruption were evaluated in sensitivity analyses, in which the minimum duration of the interruption was increased to ≥ 60 or ≥ 90 days. These represented a subset of individuals initially identified as having experienced a treatment interruption of ≥ 45 days. All analyses were repeated within each of these populations.

## Results

### Study population

Out of 76,883 people with HIV for whom an oral ART treatment interruption could be observed, 8,550 individuals (11%) experienced ≥ 1 treatment interruption of ≥ 45 days without any ART medication, based on supply from last prescription, during the eligibility period. Of these, 4,163 (49%) individuals resumed treatment during the eligibility period; these individuals formed the study population and represented 5% of people who were active in care and receiving an eligible oral ART regimen. An inclusion criteria schematic diagram can be found in Fig. 1. For most treatment interruptions (*n* = 3,151; 61%), the individual who resumed treatment reached study end on the post-interruption regimen (29FEB2024); the median follow-up time after resuming treatment was 11 months (IQR: 6, 16). Reasons for censoring included death (1%), loss to follow-up (8%), and regimen discontinuation or additional interruption (30%).

### Characteristics of treatment interruptions

Among 4,163 individuals who experienced a treatment interruption and resumed treatment during the eligibility period, there were 5,194 interruptions of ≥ 45 days that were followed by resumption. This resulted in a median of 1 treatment interruption with resumption per person (IQR: 1, 1) and a mean of 1.2 interruptions with resumption per person (range: 1, 5); 83% experienced only one treatment interruption with resumption during the eligibility period. The median duration of a treatment interruption with resumption was 100 days (IQR: 67, 166) while the mean duration was 135 days (standard deviation [sd]: 103). In each of the first two years of the eligibility period (01JUL2021–30JUN2022 and 01JUL2022–30JUN2023), 3% of eligible individuals experienced a treatment interruption.

### Baseline demographic and clinical characteristics

The median age of the study population was 44 years (IQR: 33, 56) and 81% were male (Table 1). There were 102 individuals (2%) who identified as transgender. The study population was diverse with 52% of individuals identifying as Black, 41% as White, and 18% as Hispanic. The majority were using commercial insurance (54%) but Medicaid (35%) was also common. Pregnancy was rare, with only 6 pregnancies within 4.5 months of the start of a treatment interruption, representing 0.1% of treatment interruptions. Median time since HIV diagnosis was 118 months (IQR: 54, 217).

**Table 1:** Characteristics of people with HIV who experienced a treatment interruption of ≥ 45 days with resumption of treatment during the eligibility period

	*N* = 4,163
Median age in years (IQR)	44 (33, 56)
Female, n (%)	777 (19)
Race, n (%)	
Asian	56 (1)
Black	2,159 (52)
White	1,716 (41)
Other/Unknown	232 (6)
Hispanic ethnicity, n (%)	762 (18)
US Geographic Region, n (%)	
Northeast	1,315 (32)
South	2,167 (52)
Midwest	80 (2)
West	571 (14)
US Territories	30 (1)
Payer^a^, n (%)	
Medicaid	1,443 (35)
Medicare	686 (16)
Commercial Insurance	2,266 (54)
ADAP/Ryan White	942 (23)
Other^b^	1,193 (32)
Months since HIV diagnosis, median (IQR)	118 (54, 217)
Any comorbid condition listed below, n (%)	3,288 (79)
Cardiovascular disease	528 (13)
Invasive cancer	297 (7)
Endocrine disorders	1,777 (43)
Mental health conditions	2,121 (51)
Liver disease	785 (19)
Bone disorders	197 (5)
Renal disease or impairment	687 (17)
Autoimmune disorders	135 (3)
Substance use disorder	1,063 (26)

ADAP, AIDS Drug Assistance Program; IQR, interquartile range; n, number

^a^ Payer categories are not mutually exclusive

^b^ Includes cash, any type of insurance not listed, and unknown

Comorbidities were common in the study population with 79% ever having a comorbidity, including a mental health condition (51%), endocrine disorder (43%), or substance use disorder (26%; Table 1). Healthcare resource utilization varied widely within the study population. Among 3,874 individuals who had received care in OPERA for ≥ 12 months prior to treatment interruption, the median number of visits in the last 12 months was 6 (IQR: 1, 15) while the mean number of visits was 11 (sd: 16).

### Pre- and post-interruption ART regimen

Individuals had been on their pre-interruption regimen for a median 13 months (IQR: 4, 13) before they experienced a treatment interruption. Of pre-interruption regimens, 85% were INSTI-based, including 49% (*n* = 2,564) that were B/F/TAF, specifically. Other common anchor agents included dolutegravir (24%), darunavir (11%), elvitegravir (10%), and rilpivirine (10%). No other anchor agent surpassed 2% frequency.

For nearly all treatment interruptions (92%) with resumption, treatment was resumed with identical anchor agents to their interrupted regimen (Table 2). Regimen switches comprised of simplification (33%), anchor agent class switch (21%), anchor agent switch within a class (45%), and addition of anchor agent (20%). B/F/TAF was the post-interruption regimen used at treatment resumption in the majority (51%; *n* = 2,671) of treatment interruptions. Notably, of the 2,564 B/F/TAF interruptions, 2,489 (97%) resumed with B/F/TAF.

**Table 2:** Post-interruption regimen characteristics after a treatment interruption of ≥ 45 days, among interruptions with resumed treatment

	*N* = 5,194
Months from start of post-interruption regimen to censoring, median (IQR)	11 (6, 16)
Identical anchor agents on pre- and post-interruption regimen, n (%)	4,763 (92)
Different anchor agents on pre- and post- interruption regimen, n (%)	431 (8)
Type of change from pre- to post-interruption regimen, n (%)^a^	
Simplification^b^	144 (33)
Anchor agent class switch^c^	92 (21)
Anchor agent switch within a class	193 (45)
Addition of anchor agent	86 (20)
Post-interruption regimen included long-acting injectable ART, n (%)^d^	18 (< 1)
Post-interruption regimen consisted of bictegravir, emtricitabine, and tenofovir alafenamide, n (%)	2,671 (51)
Pre-interruption regimen consisted of bictegravir, emtricitabine, and tenofovir alafenamide, n (%)	2,489 (93)

ART, antiretroviral therapy; IQR, interquartile range; n, number

^a^ Categories are not mutually exclusive

^b^ Reduction in pill count, fewer anchor agents, switch from oral regimen to CAB + RPV LA

^c^ Replacement of an anchor agent class with a different anchor agent class

^d^ CAB + RPV LA or LEN LA

### Virologic outcomes

Virologic outcomes were only investigated among individuals who resumed treatment with B/F/TAF, the most common post-interruption regimen (51%). There was a viral load measurement available within 6 months before the start of the treatment interruption for 1,490 (56%) treatment interruptions with resumption of B/F/TAF. Of these, 1,322 (85%) were virologically suppressed (viral load < 200 copies/mL) and 1,164 (78%) were virologically undetectable (viral load < 50 copies/mL) at the measurement nearest to the beginning of the treatment interruption.

At the time of treatment resumption with B/F/TAF, viral load measurements were available within 4 weeks of resuming treatment for 1,923 (72%) interruptions. Most (85%) viral loads available at resumption were measured on or before the date of resumption of treatment, and 7% were within one week after resuming treatment (Table S1). There were 500 (26%) treatment interruptions for which the viral load measurement was viremic at start of post-interruption regimen. Of these, virologic suppression was achieved for 255 (51%) interruptions over a median time of 3 months (IQR: 1, 5) of follow-up. For these interruptions, the cumulative probability of achieving virologic suppression on B/F/TAF was 44% through 6 months, 54% through 12 months, and 68% over all available follow-up (Table 3). Please refer to Table S2 for viral load availability and measurements at various time points.

**Table 3:** Virologic outcomes after resumption with bictegravir/emtricitabine/tenofovir Alafenamide following a treatment interruption of ≥ 45 days

	*N* = 1,923
Viremic^a^ at start of post-interruption regimen, n (%)	500 (26)
Achieved virologic suppression^b^ over follow-up, n (%)	255 (51)
Months from start of post-interruption regimen to virologic suppression, median (IQR)	3 (1, 5)
Cumulative probability of virologic suppression at time points	
6 months	
At risk, n	265
Cumulative probability, % (95% CI)	44 (39, 48)
12 months	
At risk, n	84
Cumulative probability, % (95% CI)	54 (49, 58)
Over all available follow-up, % (95% CI)	68 (57, 78)
Virologically suppressed at start of post-interruption regimen, n (%)	1,423 (74)
Experienced virologic failure^c^ over follow-up, n (%)	22 (2)
Months from start of post-interruption regimen to virologic failure, median (IQR)	9 (5, 14)
Cumulative probability of virologic failure	
6 months	
At risk, n	1,128
Cumulative probability, % (95% CI)	1 (0, 1)
12 months	
At risk, n	610
Cumulative probability, % (95% CI)	1 (1, 2)
Over all available follow-up, % (95% CI)	3 (2, 5)

CI, confidence interval; IQR, interquartile range; n, number

^a^ Viremic: viral load ≥ 200 copies/mL

^b^ Virologic suppression: viral load < 200 copies/mL

^c^ Virologic failure was defined as either: (1) two consecutive viral loads ≥ 200 copies/mL or (2) change in core agent following a viral load ≥ 200 copies/mL

When the viral load measurement was suppressed (viral load < 200 c/mL) at resumption (*n* = 1,423; 74%), 22 (2%) virologic failures were observed with a median of 9 months (IQR: 5, 14) since resumption of treatment. The cumulative probability of virologic failure was 3% through all available follow-up (Table 3)

### Sensitivity analyses

Out of 76,883 eligible individuals, 7,697 (10%) experienced a ≥ 60-day gap in treatment and 3,613 (5%) experienced a ≥ 60-day treatment interruption with resumption. Further, out of 76,883 eligible individuals, 6,090 (8%) experienced a ≥ 90-day gap in treatment and 2,687 (3%) experienced a ≥ 90-day treatment interruption with resumption. The characteristics, ART regimens, and virologic outcomes of PWH who experienced ≥ 1 treatment interruption of ≥ 60 and ≥ 90 days were similar to the larger group who experienced ≥ 1 treatment interruption of ≥ 45 days (Tables S2 and S3).

## Discussion

In this real-world investigation of oral ART treatment interruptions among people with HIV in routine clinical care in the United States, 11% of individuals in OPERA for whom a treatment interruption could be observed experienced at least one treatment interruption of ≥ 45 days and 5% (49% of those with ≥ 1 interruption) resumed treatment during the eligibility period. Of these, most individuals (83%) had only one interruption with resumption. The most common ART regimen before and after treatment interruptions was B/F/TAF. After treatment interruption, individuals who resumed with B/F/TAF were generally successful in achieving virologic suppression or avoiding virologic failure. Indeed, most individuals who resumed treatment with B/F/TAF were virologically suppressed at resumption of treatment and the cumulative probability of virologic failure over all follow-up was only 3% when the viral load measurement at resumption of treatment was suppressed, highlighting the forgiving nature of modern ART. When the viral load measurement at resumption of treatment was viremic, the cumulative probability of achieving virologic suppression over all follow-up was 68% with a median time of only three months.

Prior studies of unplanned treatment interruptions have been inconsistent with respect to their definition. One systematic review [29] focusing on 70 studies from 1996 to 2011 found that most studies did not mention a specific duration and, among those that did, the duration ranged from ≥ 1 day to ≥ 1 year. Further, it was determined that 23% of patients in these reviewed studies experienced a treatment interruption, and that interruptions lasted a median of 150 days. Often, these studies relied on self-reported treatment interruptions but sometimes used clinical records, prescription history, or EHR data. Recent studies have highlighted treatment interruptions as potentially more common. For instance, one study of people in the US on Medicare from 2013 to 2018 found that 55% of individuals had a treatment gap of ≥ 7 days and 26% had a treatment gap of ≥ 30 days [30]. However, the frequency of treatment interruptions across studies is not consistent across region or duration of treatment interruption [29, 31, 32]. Therefore, a strength of the present study is that multiple thresholds for interruption periods were examined in the same study population (≥ 45, ≥ 60, and ≥ 90 days). Interestingly, individuals who had different minimum interruption durations were both similar at baseline and experienced similar virologic outcomes over follow-up. It should be noted that they are subsets of the population who experienced a treatment interruption of ≥ 45 days, representing 87% and 65% of that population, respectively. Although studies may vary in their definition of treatment interruption, they agree with the present study that treatment interruptions are common in care of people with HIV, highlighting the importance of better understanding their frequency, risk factors, and effects on virologic outcomes. While the estimate of the frequency of treatment interruptions in the present study is lower than what has been observed elsewhere, the shorter study period of 26 months and potential for loss to follow-up could explain this difference. Further highlighting these effects, resumptions were only observed for 49% of treatment interruptions during the eligibility period.

Early real-world studies of unplanned treatment interruption have found negative effects of treatment interruptions, including increased resistance to ART and virologic failure [31, 33, 34]. However, there have been few such real-world studies in recent years. A recent review of studies from 2012 to 2018, including both planned and unplanned treatment interruptions, highlighted that treatment interruptions can be managed with only minimal impact on viral load and CD4 count [4]. This is primarily due to advances in the effectiveness and tolerability of modern ART medication. Three more recent studies from different countries in Africa found unfavorable outcomes, including a slower return to normal CD4 cell counts, suboptimal treatment outcomes, and increased risk of all-cause mortality after treatment interruptions [32, 35, 36]. Accordingly, a study in Indonesia found that only having a single treatment interruption, as opposed to multiple prior interruptions, improved the likelihood of achieving a viral load < 400 copies/mL after resuming ART [37]. Findings of these studies are in alignment with the present study, suggesting that people with treatment interruptions can generally achieve virologic suppression or avoid virologic failure after restarting treatment, but that there are both immediate and long-term negative effects of the treatment interruption which can vary at the individual-level. Importantly, fewer than 50% of PWH who experienced a treatment interruption resumed treatment during the eligibility period. Additional real-world data are needed to characterize these individuals and inform targeted interventions to re-engage them into HIV care.

This study was conducted using the OPERA cohort, a large database of EHR data from clinics across the US. At the time of the study, OPERA included prospectively collected data on clinical diagnoses, prescriptions, and laboratory results of over 151,000 people with HIV, representing approximately 14% of people with HIV in the US and dependent areas [38]. This large database of routine clinical care data provided access to information on ART prescriptions and laboratory testing of a sufficient number of individuals to accomplish the goals of the study. This study was able to follow individuals longitudinally, allowing for trajectories of virologic outcomes over time to be investigated in a real-world, routine clinical care setting. Further, this study allowed for comparison of different thresholds of minimum duration of a treatment interruption, demonstrating that characteristics and outcomes were similar across these thresholds.

The study was limited by the potential for missing data, especially if an individual received care from a non-OPERA provider. As a result, information on virologic outcomes might be unavailable. This study was descriptive in nature and did not use a comparison group for the assessment of virologic outcomes among individuals who resumed treatment with B/F/TAF. Although this represented a slight majority of people who experienced at least one treatment interruption with resumption, it did not allow for comparison of outcomes by post-interruption regimen. Given that follow-up was censored at regimen discontinuation, the amount of available follow-up time after each treatment interruption with resumption was limited. However, each treatment interruption for a given individual during the eligibility period was included in the analysis. While this approach maximized the number of treatment interruptions observed, it could potentially lead to within-subject correlation of HIV viral load data observed over follow-up for the 17% of individuals who experienced more than one treatment interruption during the eligibility period. Availability of viral load measurements at start of treatment interruption and upon resumption of treatment was also limited. To account for this, a window of up to 6 months before the start of interruption was used to describe the period leading up to the interruption. Upon resumption of treatment, a window of ± 4 weeks was considered to maximize the number of individuals for which virologic outcomes over follow-up could be investigated. Of the viral load measurements at this point, 85% were measured on or before the date of resumption while only 9% were more than one week after resuming treatment. Additionally, resistance testing data were not available to identify reasons for discontinuation or characterization of individuals who were viremic when resuming treatment. When available, resistance testing and ART history can be useful in selecting an ART regimen.

As with many studies relying on EHR data to determine ART use, there is a level of uncertainty around regimen stop dates, reasons for regimen change, date of refills, use of medication samples, and potential stockpiling of excess pills over time. While algorithms have been established and tested to mitigate this limitation, these pitfalls could limit our ability to assess treatment adherence. Given these limitations, a definition of ≥ 45 days without treatment was considered as a treatment interruption while sensitivity analyses of ≥ 60 and ≥ 90 days were also used. Among all three definitions, similar characteristics, ART regimens, and virologic outcomes over follow-up were observed likely due to the generally long duration (mean: 135 days; sd: 103 days) of treatment interruptions in the study. Because of this, future work could consider the effect of longer interruptions, specifically.

## Conclusions

During the 26-month study period, treatment interruptions of ≥ 45 days with resumption of treatment occurred in 11% of ART users in routine clinical care, representing a large number of people at heightened risk for loss of virologic control, reduced immune function, selection of resistance, and increased likelihood of transmitting HIV to others. After resuming treatment with B/F/TAF, a majority of individuals were able to achieve virologic suppression or avoid virologic failure, affirming it as an option when optimal adherence may be a challenge.

## Electronic supplementary material

Below is the link to the electronic supplementary material.

Supplementary Material 1

## Data Availability

The datasets used in this study are not publicly available due to privacy concerns and the proprietary nature of the database but can be accessed upon reasonable request through the corresponding author to the OPERA Epidemiological and Clinical Advisory Board. Access to codes may be granted upon request with parties agreeing to privacy restrictions and technological specifications and requirements.

## Abbreviations

ART: Antiretroviral therapy
ARV: Antiretroviral
B/F/TAF: Bictegravir/emtricitabine/tenofovir alafenamide
EHR: Electronic health records
HIPAA: Health Insurance Portability and Accountability Act
HITECH: Health Information Technology for Economic and Clinical Health Act
HIV: Human Immunodeficiency Virus
IQR: Interquartile range
IRB: Institutional Review Board
OPERA^®^: Observational Pharmaco-Epidemiology Research and Analysis
n: Number
PWH: People with HIV
sd: Standard deviation
US: United States
