# Supplementary material for: Characterizing treatment interruptions in the OPERA cohort and virologic outcomes after resumption with bictegravir/emtricitabine/tenofovir alafenamide
Source: AIDS Res Ther. 2025 Jul 21;22:71. doi: 10.1186/s12981-025-00769-x (PMC12281873; doi:10.1186/s12981-025-00769-x)
Supplement: Supplementary file 1 — Supplementary Material 1 [file 12981_2025_769_MOESM1_ESM.docx]

**Supplementary Material**

Table S1. Timing of nearest available viral load measurement to time of resumption of treatment, among treatment interruptions resumed with bictegravir/emtricitabine/tenofovir alafenamide

|  | **N = 1,923** |
| --- | --- |
| Days from resumption of treatment, median (IQR)^a^ | 0 (-1, 0) |
| Days from resumption of treatment, mean (sd)^a^ | -1 (8) |
| Time from resumption of treatment, n (%) |  |
| Before (up to 28 days) or on date of resumption of treatment | 1,631 (85) |
| 1 to 7 days after resuming treatment | 134 (7) |
| 8 to 14 days after resuming treatment | 55 (3) |
| 15 to 21 days after resuming treatment | 59 (3) |
| 22 to 28 days after resuming treatment | 54 (3) |

*IQR, interquartile range; n, number; sd, standard deviation*

^a^ Days before resumption of treatment are counted as negative numbers

Table S2. HIV viral load measurements at various time points, by minimum duration of treatment interruption, among treatment interruptions resumed with bictegravir/emtricitabine/tenofovir alafenamide

|  | **Treatment interruptions**  **of ≥45 days N = 2,671** | **Treatment interruptions**  **of ≥60 days**  **N = 2,252** | **Treatment interruptions**  **of ≥90 days**  **N = 1,458** |
| --- | --- | --- | --- |
| HIV viral load available within 6 months before/at start of treatment interruption, n (%) | 1,490 (56) | 1,221 (54) | 787 (49) |
| HIV viral load (copies/mL) within 6 months before/at start of treatment interruption, median (IQR) | 19 (19, 32) | 19 (19, 39) | 19 (19, 40) |
| ≥1,000, n (%) | 129 (9) | 109 (9) | 81 (10) |
| ≥200 to <1,000, n (%) | 39 (3) | 32 (3) | 26 (3) |
| ≥50 to <200, n (%) | 158 (11) | 132 (11) | 74 (9) |
| <50, n (%) | 1,164 (78) | 948 (78) | 606 (77) |
| HIV viral load available at start of post-interruption ART regimen (±4 weeks), n (%) | 1,923 (72) | 1,628 (72) | 1,154 (72) |
| HIV viral load (copies/mL) at start of post-interruption ART regimen (±4 weeks), median (IQR) | 20 (19, 231) | 20 (19, 465) | 20 (19, 3460) |
| ≥1,000, n (%) | 421 (22) | 381 (23) | 325 (28) |
| ≥200 to <1,000, n (%) | 79 (4) | 68 (4) | 49 (4) |
| ≥50 to <200, n (%) | 200 (10) | 169 (10) | 120 (10) |
| <50, n (%) | 1,223 (64) | 1,010 (62) | 660 (57) |
| HIV viral load available 3 months after start of post-interruption ART regimen (±4 weeks), n (%) | 634 (24) | 541 (24) | 358 (22) |
| HIV viral load (copies/mL) 3 months after start of post-interruption ART regimen (±4 weeks), median (IQR) | 19 (19, 30) | 19 (19, 30) | 19 (19, 40) |
| ≥1,000, n (%) | 49 (8) | 44 (8) | 33 (9) |
| ≥200 to <1,000, n (%) | 15 (2) | 12 (2) | 9 (3) |
| ≥50 to <200, n (%) | 68 (11) | 56 (10) | 44 (12) |
| <50, n (%) | 502 (79) | 429 (79) | 272 (76) |
| HIV viral load available 6 months after start of post-interruption ART regimen (±4 weeks), n (%) | 552 (21) | 487 (22) | 333 (21) |
| HIV viral load (copies/mL) 6 months after start of post-interruption ART regimen (±4 weeks), median (IQR) | 19 (19, 40) | 19 (19, 40) | 19 (19, 36) |
| ≥1,000, n (%) | 49 (9) | 43 (9) | 29 (9) |
| ≥200 to <1,000, n (%) | 21 (4) | 19 (4) | 11 (3) |
| ≥50 to <200, n (%) | 58 (11) | 54 (11) | 34 (10) |
| <50, n (%) | 424 (77) | 371 (76) | 259 (78) |
| HIV viral load available 12 months after start of post-interruption ART regimen (±4 weeks), n (%) | 314 (12) | 268 (12) | 185 (12) |
| HIV viral load (copies/mL) 12 months after start of post-interruption ART regimen (±4 weeks), median (IQR) | 19 (19, 30) | 19 (19, 23) | 19 (19, 22) |
| ≥1,000, n (%) | 26 (8) | 23 (9) | 15 (8) |
| ≥200 to <1,000, n (%) | 7 (2) | ≤5 | ≤5 |
| ≥50 to <200, n (%) | 22 (7) | 17 (6) | 15 (8) |
| <50, n (%) | 259 (82) | 223 (83) | 154 (83) |

*ART, antiretroviral; IQR, interquartile range; n, number*

Table S3. Baseline characteristics of individuals who experienced a treatment interruption with resumption, by minimum duration of treatment interruption for inclusion

|  | **PWH who experienced a treatment interruption of ≥45 days**  **N = 4,163** | **PWH who experienced a treatment interruption of ≥60 days^a^**  **N = 3,613** | **PWH who experienced a treatment interruption of ≥90 days^a^**  **N = 2,687** |
| --- | --- | --- | --- |
| Median age in years (IQR) | 44 (33, 56) | 43 (33, 55) | 42 (33, 55) |
| Female, n (%) | 777 (19) | 678 (19) | 509 (19) |
| Race, n (%) |  |  |  |
| Asian | 56 (1) | 49 (1) | 34 (1) |
| Black | 2,159 (52) | 1,883 (52) | 1,389 (52) |
| White | 1,716 (41) | 1,488 (41) | 1,120 (42) |
| Other/Unknown | 232 (6) | 193 (5) | 144 (5) |
| Hispanic ethnicity, n (%) | 762 (18) | 674 (19) | 513 (19) |
| US Geographic Region, n (%) |  |  |  |
| Northeast | 1,315 (32) | 1,158 (32) | 843 (31) |
| South | 2,167 (52) | 1,874 (52) | 1,400 (52) |
| Midwest | 80 (2) | 71 (2) | 55 (2) |
| West | 571 (14) | 487 (13) | 371 (14) |
| US Territories | 30 (1) | 23 (1) | 18 (1) |
| Payer^b^, n (%) |  |  |  |
| Medicaid | 1,443 (35) | 1,255 (35) | 936 (35) |
| Medicare | 686 (16) | 596 (17) | 416 (15) |
| Commercial Insurance | 2,266 (54) | 1,948 (54) | 1,411 (53) |
| ADAP/Ryan White | 942 (23) | 815 (23) | 588 (22) |
| Other^c^ | 1,193 (32) | 1040 (29) | 780 (29) |
| Months since HIV diagnosis, median (IQR) | 118 (54, 217) | 118 (54, 217) | 116 (54, 213) |
| Any comorbid condition listed below, n (%) | 3,288 (79) | 2,841 (79) | 2,083 (78) |
| Cardiovascular disease | 528 (13) | 447 (12) | 311 (12) |
| Invasive cancer | 297 (7) | 238 (7) | 170 (6) |
| Endocrine disorders | 1,777 (43) | 1,538 (43) | 1,099 (41) |
| Mental health conditions | 2,121 (51) | 1,840 (51) | 1,350 (50) |
| Liver disease | 785 (19) | 692 (19) | 499 (19) |
| Bone disorders | 197 (5) | 168 (5) | 118 (4) |
| Renal disease or impairment | 687 (17) | 592 (16) | 415 (15) |
| Autoimmune disorders | 135 (3) | 114 (3) | 85 (3) |
| Substance use disorder | 1,063 (26) | 932 (26) | 714 (27) |

*ADAP, AIDS Drug Assistance Program; IQR, interquartile range; n, number; PWH, people with HIV*

^a^ PWH who experienced a treatment interruption of ≥60 days and ≥90 days are a subset of PWH who experienced a treatment interruption of ≥45 days

^b^ Payer categories are not mutually exclusive

^c^ Includes cash, any type of insurance not listed, and unknown
